# Deep Learning for Genomics: From Early Neural Nets to Modern Large Language Models

**DOI:** 10.3390/ijms242115858

**Published:** 2023-11-01

**Authors:** Tianwei Yue, Yuanxin Wang, Longxiang Zhang, Chunming Gu, Haoru Xue, Wenping Wang, Qi Lyu, Yujie Dun

**Affiliations:** 1 School of Computer Science, Carnegie Mellon University, Pittsburgh, PA 15213, USA; yuanxinw@alumni.cmu.edu (Y.W.); longxiaz@alumni.cmu.edu (L.Z.); wenpingw@alumni.cmu.edu (W.W.); 2 Department of Biomedical Engineering, School of Medicine, Johns Hopkins University, Baltimore, MD 21218, USA; cgu15@jhmi.edu; 3 The Robotics Institute, Carnegie Mellon University, Pittsburgh, PA 15213, USA; haorux@andrew.cmu.edu; 4 Department of Computational Mathematics, Science, and Engineering, Michigan State University, East Lansing, MI 48824, USA; lyuqi1@msu.edu; 5 School of Information and Communications Engineering, Xi’an Jiaotong University, Xi’an 710049, China; dunyj@mail.xjtu.edu.cn

**Keywords:** deep learning, genomics, large language model, computer vision, multi-modal machine learning

## Abstract

The data explosion driven by advancements in genomic research, such as high-throughput sequencing techniques, is constantly challenging conventional methods used in genomics. In parallel with the urgent demand for robust algorithms, deep learning has succeeded in various fields such as vision, speech, and text processing. Yet genomics entails unique challenges to deep learning, since we expect a superhuman intelligence that explores beyond our knowledge to interpret the genome from deep learning. A powerful deep learning model should rely on the insightful utilization of task-specific knowledge. In this paper, we briefly discuss the strengths of different deep learning models from a genomic perspective so as to fit each particular task with proper deep learning-based architecture, and we remark on practical considerations of developing deep learning architectures for genomics. We also provide a concise review of deep learning applications in various aspects of genomic research and point out current challenges and potential research directions for future genomics applications. We believe the collaborative use of ever-growing diverse data and the fast iteration of deep learning models will continue to contribute to the future of genomics.

## 1. Introduction

Even since Watson and Crick [1] first interpreted DNA molecules as the physical medium carrying genetic information, human beings have been striving to gather biological data and decipher the biological processes guided by genetic information. By 2001, the Human Genome Project launched in 1990 had drafted the raw information of a typical human genome [2]. Many other genome projects, including FANTOM [3], ENCODE [4], and Roadmap Epigenomics [5], were also launched in succession. These collaborative efforts made an abundance of DNA data available and thus allowed a global perspective on the genome of different species, leading to the prosperity of genomic research.

Genomic research aims to understand the genomes of different species. It studies the roles assumed by multiple genetic factors and the way they interact with the surrounding environment under different conditions. In contrast to genetics, which deals with a limited number of specific genes, genomics takes a global view that involves the entirety of genes possessed by an organism [6]. For example, a study of homo sapiens involves searching through approximately 3 billion units of DNA, containing protein-coding genes, RNA genes, cis-regulatory elements, long-range regulatory elements, and transposable elements [7]. Additionally, genomics is becoming increasingly data intensive with the advancement in genomic research, such as the cost-effective next-generation sequencing technology that produces the entire readout of the DNA of an organism. This high-throughput technology is made available by more than 1000 sequencing centers cataloged by OmicsMaps (http://omicsmaps.com/ (accessed on 3 September 2023)) on nearly every continent [8]. The vast trove of information generated by genomic research provides a potential exhaustive resource for scientific study with statistical methods. These statistical methods can be used to identify different types of genomic elements, such as exons, introns, promoters, enhancers, positioned nucleosomes, splice sites, untranslated regions (UTRs), etc. In addition to recognizing these patterns in DNA sequences, models can take other genetic and genomic information as input to build systems to help understand the biological mechanisms of underlying genes. A large variety of data types are available, such as chromatin accessibility assays (e.g., MNase-seq, DNase-seq, FAIRE), genomic assays (e.g., microarray, RNA-seq expression), transcription factor (TF)-binding ChIP-seq data, gene expression profiles, histone modifications, etc. [9]. Most of these data are available through portals like GDC (https://portal.gdc.cancer.gov/ (accessed on 15 September 2023)), dbGaP (https://www.ncbi.nlm.nih.gov/gap (accessed on 15 September 2023)), and GEO (https://www.ncbi.nlm.nih.gov/geo/ (accessed on 15 September 2023)), just to name a few. A combination of various data can bring about deeper insights into genes so as to help researchers locate the information of interest.

On the other hand, the development of deep learning methods has granted the computational power to resolve these complex research questions [10,11]. Its success has already been demonstrated by the revolutionizing achievements in the field of artificial intelligence, e.g., image recognition, object detection, audio recognition, natural language processing, etc. The boom of deep learning is supported by the successive introduction of a variety of deep architectures, including autoencoders [12] and their variants, multilayer perceptron (MLP; [13,14]), restricted Boltzmann machines (RBMs; [15]), deep belief networks (DBNs; [16]), convolutional neural networks (CNNs; [17,18]), recurrent neural networks (RNNs; [19]), Long Short-Term Memory (LSTM; [20]), Transformers [21], large language models [22,23,24], and other recently appearing architectures that will be introduced later in this article. The strong flexibility and high accuracy of deep learning methods guarantee them sweeping superiority over other existing methods on these classical tasks.

The intersection of deep learning methods and genomic research may lead to a profound understanding of genomics that will benefit multiple fields including precision medicine [25], pharmacy (i.e., drug design), and even agriculture. Take medicine, for example: medical research and its applications such as gene therapies, molecular diagnostics, and personalized medicine could be revolutionized by tailoring high-performance computing methods to analyze available genomic datasets. Also, the process of developing new drugs takes a long period and is usually very costly. To save time and cost, the general approach taken by pharmaceutical companies is to try to match the candidate protein identified by researchers with their known drug molecules [26]. As we are facing larger-scale and more complex medical demands, cutting-edge deep learning techniques such as large language models show emergent capabilities to efficiently and effectively deal with unprecedented challenges such as COVID-19 [27,28]. In addition, deep learning techniques have also facilitated research in survival predictions and subtype classifications in lung cancer [29]. All these benefits indicate the necessity of utilizing powerful and specially designed deep learning methods to foster the development of the genomics industry. This article aims to offer a concise overview of the current deep learning applications in genomic research and, if possible, point out promising directions for further applying deep learning in the genomic study.

The rest of this article is organized as follows: we first briefly introduce the genomic study powered by deep learning characterized by deep learning architectures in Section 2 with additional discussions offered in Section 3. Then, we discuss the use of deep learning methods on various topics in genomics in Section 4, which is followed by our summarization of the current challenges and potential research directions in Section 5. Finally, conclusions are drawn in Section 6.

## 2. Deep Learning Architectures: A Genomic Perspective

Various deep learning algorithms have their own advantages to resolve particular types of problems in genomic applications (see a comprehensive list in Table 1). For example, CNNs that are famous for capturing features in image classification tasks have been widely adopted to automatically learn local and global characterizations of genomic data. RNNs that succeed in speech recognition problems are skillful at handling sequence data and thus were mostly used to deal with DNA sequences. Autoencoders are popular for both pre-training models and denoising or pre-processing the input data. LLMs are known for their emergent capabilities in dealing with extremely long-range interactions in sequences. When designing deep learning models, researchers could take advantage of these merits to efficiently extract reliable features and reasonably model the biological process. For example, with sufficient labeled data, traditional CNNs and RNNs might be used as solid baselines; when robust representations are needed for various downstream tasks, VAEs could be a good point to start; if the capability of coping with long input sequences is required, LLMs should come into play. This section will review some details on each type of deep architecture (deep learning-based architecture), focusing on how each of their advantages can benefit the specific genomic research questions. This article will not cover the standard introduction of deep learning methods; readers can visit classical textbooks, e.g., [30] or concise tutorials, e.g., [11] if necessary.

### 2.1. Convolutional Neural Networks

Convolutional neural networks (CNNs) are one of the most successful deep learning models for image processing owing to their outstanding capacity to analyze spatial information. Early applications of CNNs in genomics relied on the fundamental building blocks of CNNs in computer vision [79] to extract features. Zeng et al. [34] described the adaptation of CNNs from the field of computer vision to genomics as accomplished by comprehending a window of genome sequence as an image.

The highlight of CNNs is the dexterity of automatically performing adaptive feature extraction during the training process. For instance, CNNs can be applied to discover meaningful recurring patterns with small variances, such as genomic sequence motifs. This makes CNNs suitable for motif identification and therefore binding classification [35].

Recently, CNNs have been shown to take a lead among current algorithms for solving several sequence-based problems. Alipanahi et al. [31], DeepBind, and [34] successfully applied CNNs to model the sequence specificity of protein binding. Zhou and Troyanskaya [32] (DeepSEA) developed a conventional three-layer CNN model to predict from only genomic sequence the effects of noncoding variants. Kelley et al. [36] and Basset adopted a similar architecture to study the functional activities of DNA sequences.

Although multiple researchers have demonstrated the superiority of CNNs over other existing methods, inappropriate structure design would still result in even poorer performance than conventional models. For example, Zeng et al. [34] conducted a comprehensive analysis of CNN networks of various architectures on the task of motif discovery and motif occupancy in genomic sequences, and they showed that although an increasing number of convolutional kernels generally increases model performance, the performance may be indifferent or even negatively impacted by an increasing number of convolutional layers and inappropriate pooling methods. Therefore, what remains is for researchers to master and optimize the ability of CNNs to skillfully match a CNN architecture to each particular given task. To achieve this, researchers should have an in-depth understanding of CNN architectures as well as take into consideration the biological background. Zeng et al. [34] developed a parameterized convolutional neural network to conduct a systematic exploration of CNNs on two classification tasks, motif discovery, and motif occupancy. They performed a hyper-parameter search using Mri (https://github.com/Mri-monitoring/Mri-docs/blob/master/mriapp.rst (accessed on 14 September 2023)) and mainly examined the performance of nine variants of CNNs, and they concluded that CNNs do not need to be deep for motif discovery tasks as long as the structure is appropriately designed. When applying CNNs in genomics, simply changing the network depth would not account for much improvement in model performance. This is because deep learning models are usually over-parameterized, meaning there are more parameters in the neural network than what is actually required to complete the task [80]. In this direction, Xuan et al. [44] designed a dual CNN with attention mechanisms to extract deeper and more complex feature representations of lncRNA (long noncoding RNA genes); while Kelley et al. [43,45] took a different path in using dilated convolution instead of classical convolution to share information across long distances without adding depth indefinitely.

### 2.2. Recurrent Neural Networks

Recurrent neural networks (RNNs) raised a surge of interest owning to their impressive performance on sequential prediction problems such as language translation, summarization, and speech recognition. RNNs outperform CNNs and other early deep neural networks (DNNs) on sequential data thanks to their capability of processing long ordered sequences and memorizing long-range information through recurrent loops. Specifically, RNNs scan the input sequences sequentially and feed both the previously hidden layer and current input segment as the model input so that the final output implicitly integrates both current and previous information in the sequence. Schuster and Paliwal [81] later proposed bidirectional RNN (BRNN) for use cases where both past and future contexts in the input matter.

The cyclic structure makes a seemingly shallow RNN over long-time prediction actually very deep if unrolled in time. To resolve the vanishing gradient problem rendered by this, Hochreiter and Schmidhuber [20] substituted the hidden units in RNNs with LSTM units to truncate the gradient propagation. Cho et al. [82] introduced Gated Recurrent Units (GRUs) with a similar proposal.

Genomics data are typically sequential and often considered languages of biological nature. Recurrent models are thus applicable in many scenarios. For example, Cao et al. [50] (ProLanGO) built an LSTM-based Neural Machine Translation, which converts the task of protein function prediction to a language translation problem by interpreting protein sequences as the language of Gene Ontology terms. Boža et al. [52] developed DeepNano for base calling, Quang and Xie [49] proposed DanQ to quantify the function of noncoding DNA, Sønderby et al. [48] devised a convolutional LSTM to predict protein subcellular localization from protein sequences, Busia et al. [73] applied the idea of seq-to-seq learning to their model for protein secondary structure prediction conditioned on previously predicted labels, and Wang et al. [83] used bidirectional LSTM (Bi-LSTM) in their prPred-DRLF predictor for plant resistance protein detection, demonstrating effective crossovers between natural language processing (NLP) and genomics [84]. Furthermore, sequence-to-sequence learning for genomics is boosted by attention mechanisms: Singh et al. [53] introduced an attention-based approach where a hierarchy of multiple LSTM modules are used to encode input signals and model how various chromatin marks cooperate; similarly, Shen et al. [85] used LSTM as a feature extractor and attention modules as importance scoring functions to identify regions of the RNA sequence that bind to proteins.

### 2.3. Autoencoders

Autoencoders, conventionally used as pre-processing tools to initialize the network weights, have been extended to stacked autoencoders (SAEs; [86]), denoising autoencoders (DAs; [87]), contractive autoencoders (CAEs; [88]), etc. Now they have proved successful in feature extraction because of being able to learn a compact representation of input through the encode–decode procedure. For example, Gupta et al. [89] applied stacked denoising autoencoders (SDAs) for gene clustering tasks. They extracted features from data by forcing the learned representation resistant to a partial corruption of the raw input. More examples can be found in Section 4.1.1. Autoencoders are also also used for dimension reduction in gene expression, e.g., [90,91,92]. When applying autoencoders, one should be aware that better reconstruction accuracy does not necessarily lead to model improvement [93].

Variational autoencoders (VAEs), though named “autoencoders”, were rather developed as an approximate-inference method to model latent variables. Based on the structure of autoencoders, Kingma and Welling [94] added stochasticity to the encoded units and added a penalty term encouraging the latent variables to produce a valid decoding. VAEs aim to deal with the problems in which each datum has a corresponding latent representation and are thus useful for genomic data, among which there are complex interdependencies. Rampasek and Goldenberg [93] presented a two-step VAE-based model for drug response prediction, which first predicts the post- from the pre-treatment state in an unsupervised manner and then extends it to the final semi-supervised prediction. This model was based on data from Genomics of Drug Sensitivity in Cancer (GDSC; [95]) and Cancer Cell Line Encyclopedia (CCLE; [96]). VAEs can also be used in many other genomic applications including cancer gene expression prediction [54,97], single cell feature extraction for unmasking tumor heterogeneity [56], metagenomic binning [57], DNA methylome dataset construction [55], etc.

### 2.4. Emergent Deep Architectures

As deep learning is constantly showing success in genomics, researchers are expecting deep learning to show higher accuracy than simply outperforming statistical or machine learning methods. To this end, the vast majority of work nowadays approaches genomic problems from more advanced architectures beyond classic deep architectures or employing hybrid models. Here, we review some examples of recent appearing deep architectures which skillfully modify or combine classical deep learning models.

#### 2.4.1. Beyond Classic Models

Most of these emergent advanced architectures are of natural designs modified from classic deep learning models. Researchers began to leverage more genomic intuitions to fit each particular problem with a more advanced and suitable model.

Motivated by the fact that protein folding is a progressive refinement [98] rather than an instantaneous process, Lena et al. [99] designed DST-NNs for residue–residue contact prediction. It consists of a 3D stack of neural networks in which topological structures (same input, hidden, and output layer sizes) are identical in each stack. Each level of this stacked network can be regarded as a distinct contact predictor and can be trained in a supervised matter to refine the predictions of the previous level, hence addressing the typical problem of vanishing gradients in deep architectures. The spatial features in this deep spatiotemporal architecture refer to the original model inputs, while temporal features are gradually altered so as to progress to the upper layers. Angermueller et al. [100] (DeepCpG) took advantage of two CNN sub-models and a fusion module to predict DNA methylation states. The two CNN sub-models take different inputs and thus focus on disparate purposes. The CpG module accounts for correlations between CpG sites within and across cells, while the DNA module detects informative sequence patterns (motifs). Then, the fusion module can integrate higher-level features derived from two low-level modules to make predictions. Instead of subtle modifications or combinations, some works focused on depth, trying to improve the model performance by designing even deeper architectures. Wang et al. [101] developed an ultra-DNN consisting of two deep residual neural networks to predict protein contacts from a sequence of amino acids. Each of the two residual nets in this model has its particular function. A series of 1D convolutional transformations are designed for extracting sequential features (e.g., sequence profile, predicted secondary structure, and solvent accessibility). The 1D output is converted to a 2D matrix by an operation similar to the outer product and merged with pairwise features (e.g., pairwise contact, co-evolution information, and distance potential). Then, they are together fed into the second residual network, which consists of a series of 2D convolutional transformations. The combination of these two disparate residual nets creates a novel approach that can integrate sequential features and pairwise features in one model.

#### 2.4.2. Hybrid Architectures

The fact that each type of DNN has its own strength inspires researchers to develop hybrid architectures that could well utilize the potential of multiple deep learning architectures. DanQ [49] is a hybrid convolutional and recurrent DNN for predicting the function of noncoding DNA directly from sequence alone. A DNA sequence is input as the one-hot representation of four bases to a simple convolutional neural network with the purpose of scanning motif sites. Motivated by the fact that the motifs can be determined to some extent by the spatial arrangements and frequencies of combinations of DNA sequences [49], the purported motifs learned by CNN are then fed into a Bi-LSTM. Similar convolutional-recurrent designs were further discussed by Lanchantin et al. [58] (Deep GDashboard). They demonstrated how to understand three deep architectures—convolutional, recurrent, and convolutional-recurrent networks—and verified the validity of the features generated automatically by the model through visualization techniques. They argued that a CNN–RNN architecture outperforms CNN or RNN alone based on their experimental results on a transcription factor binding site (TFBS) classification task. The feature visualization achieved by Deep GDashboard indicated that CNN–RNN architecture is able to model both motifs as well as dependencies among them. Sønderby et al. [48] added a convolutional layer between the raw data and LSTM input to address the problem of protein sorting or subcellular localization. In total, there are three types of models proposed and compared in the paper: a vanilla LSTM, an LSTM with an attention model used in a hidden layer, and an ensemble of ten vanilla LSTMs. They achieved higher accuracy than previous benchmark models in predicting the subcellular location of proteins from DNA sequences while no human-engineered features were involved. Almagro Armenteros et al. [60] proposed a hybrid integration of an RNN, a Bi-LSTM, an attention mechanism, and a fully connected layer for protein subcellular localization prediction; each of the four modules is designed for a specific purpose. These hybrid models are increasingly favored by recent research, e.g., [59].

Hybrid architectures allow flexible network design by selecting specific components with proven success representing different types of information in genomic sequences. For example, in both [49,62], CNN layers have been included to generate representations on local patterns such as regulatory motifs in DNA sequences, while RNN and attention modules are used to encode information on long-range dependence. Although hybrid architectures built on existing successful models have been proven to improve performance over single architecture, there still lacks a systematic principle or algorithm for designing or even optimizing network architecture for deep learning models in genomics.

### 2.5. Transformer-Based Large Language Models

As mentioned in Section 2.1 and Section 2.2, many prior deep learning works utilized CNNs and RNNs to solve genomics tasks. However, there are several intrinsic limitations of these two architectures. (1) CNNs might fail to capture the global understanding of a long DNA sequence due to its limited receptive field. (2) RNNs could have difficulty in capturing useful long-term dependencies because of vanishing gradients and suffer from low-efficiency problems due to their non-parallel sequence processing nature. (3) Both architectures need extensive high-quality labeled data to train. These limitations hinder them from coping with harder genomics problems since these tasks usually require the model to (1) understand long-range interactions, (2) process very long sequences efficiently, and (3) perform well even for low-resource training labels.

Transformer-based [21] language models such as BERT [102] and GPT family [22,23,24] then become a natural fit to overcome these limitations. Their built-in attention mechanism learns better representations that can be generalized to data-scarce tasks via larger receptive fields. Ref. [103] found that a pre-trained large DNA language model is able to make accurate zero-shot predictions of noncoding variant effects. Similarly, according to [104], these language model architectures generate robust contextualized embeddings on top of nucleotide sequences and achieve accurate molecular phenotype prediction even in low-data settings.

Instead of processing input tokens one by one sequentially as RNNs do, transformers process all input tokens more efficiently at the same time in parallel. However, simply increasing the input context window infinitely is infeasible, since the computation time and memory scale quadratically with context length in the attention layers. Several improvements have been made from different perspectives: Nguyen et al. [70] uses the Hyena architecture [105] and scales sub-quadratically in context length, while Zhou et al. [68] replace k-mer tokenization used in Ji et al. [63] with Byte Pair Encoding (BPE) to achieve a 3× efficiency improvement.

In light of dealing with extremely long-range interactions in DNA sequences, the Enformer model [66] employs transformer modules that scale a five times larger receptive field compared to previous CNN-based approaches [43,45,106], and it is capable of detecting sequence elements that are 100 kb away. Moreover, the recent success of ChatGPT [107] and GPT-4 [108] further illustrated the emergent capabilities of large language models (LLMs) to deal with such long DNA sequences. A typical transformer-based genomics foundational model can only take 512 to 4k tokens as input context, which is less than 0.001% of the human genome. Nguyen et al. [70] proposed an LLM-based genomic model that expands the input context length to 1 million tokens at the single nucleotide level, which is up to a 500× increase over previous dense attention-based models.

Even with all these advancements in efficiency improvement, the significant training and serving cost still remains a challenging problem for LLMs [109], especially for long input context for genomics problems. Furthermore, due to privacy concerns and legal regulations, the generation and collection of large-scale high quality genomics data usually requires complex procedures, which might slow down the iteration of model development.

## 3. Deep Learning Architectures: Insights and Remarks

Applications of deep learning in genomic problems have fully proven their power. Although the pragmatism of deep learning is surprisingly successful, this method suffers from lacking the physical transparency to be well interpreted so as to better assist the understanding of genomic problems. What is auspicious in genomic research is that researchers have conducted lots of work to visualize and interpret their deep learning models. It is also constructive to take into additional considerations beyond the choice of deep learning architectures. In this section, we review some visualization techniques that bring about insights into deep learning architectures and add remarks on model design that might be conducive to real-world applications.

### 3.1. Model Interpretation

People expect deep networks to succeed not only in predicting results but also in identifying meaningful DNA sequence signals and giving further insights into the problems being solved. The interpretability of a model appears to be crucial when it comes to application. However, the technology of deep learning has exploded not only in prediction accuracy but also in complexity as well. Connections among network units are so convoluted that the information is widespread throughout the network and thus perplexing to be captured [110]. People are carrying out efforts to remedy this pitfall, since prediction accuracy alone does not guarantee that deep architectures are a better choice over traditional statistical or machine learning methods in applications. Different visualizations techniques are also being actively developed [33,48,111,112,113].

The image classification field is where people started deciphering deep networks. Zeiler and Fergus [114] gave insights into the function of intermediate features by mapping hidden layers back to the input through deconvolution, which is a technique described in that paper. Simonyan et al. [115] linearly approximate the network by first-order Taylor expansion and obtained Saliency Maps from a ConvNet by projecting back from the dense layers of the network. People also searched for an understanding of genes through deep networks. Denas and Taylor [116] managed to pass the model knowledge back into the input space through the inverse of the activation function so that biologically meaningful patterns can be highlighted. Lanchantin et al. [58] (Dashboard) adopted Saliency Maps to measure nucleotide importance. Their work provided a series of visualization techniques to detect motifs, or sequence patterns from deep learning models, and went further to discuss the features extracted by CNNs and RNNs. Similarly, Alipanahi et al. [31] visualized the sequence specificities determined by DeepBind through mutation maps that indicate the effect of variations on bound sequences. Note that works conducted appropriately by classic models do not need additional techniques to visualize features; e.g., Pärnamaa and Parts [117] trained an 11-layer CNN for prediction protein subcellular localization from microscopy images and easily interpreted their model by features at different layers.

The rise of attention-based models also opened up new avenues for interpretability in genomics. Singh et al. [53] argued that attention scores provide a better interpretation than traditional feature visualization methods such as Saliency Maps. According to Chen et al. [118], the visualization of attention weight changes can be used to understand when binding signal peaks move along the genomic sequence. Ghotra et al. [119] further emphasized the importance of the convolutional layer(s) learning identifiable motifs for the attention maps to be interpretable.

It needs to be addressed that there still is no universal framework for an interpretability study of deep neural networks in the genomics domain. For example, although many models referred to in this paper [63,65,66,68,70,71] claim to model long-range dependence in various genomic sequences (protein or DNA), the claims were mostly only substantiated by the fact that their models can process long input sequences with a lack of quantitative alignment between long-range dependence captured by the model and interpretation through existing genomics theories. To assist DNN interpretation through the lens of genomics theory, Horel and Giesecke [120] has offered a theoretical framework for conducting significance tests on neural network parameters. Global Importance Analysis ([121], GIA) offers a versatile importance score assigned to embedded patterns in genomic sequences (DNA, RNA, protein) by calculating the difference in network output with and without a pattern in the input. Shrikumar et al. [122] (DeepLIFT) proposed a contribution score to quantify the importance of each element in the input calculated by backpropagating output variation through the network to the input. Nonetheless, these tools were studied only on one specific task: DeepLIFT was tested only on motif discovery in DNA sequences for regulatory genes, and GIA was only demonstrated on CNN networks on motif discovery tasks. It remains to be investigated if or how the visualization, attention scores, and importance measure can be adopted for a more generalized use of interpreting deep neural networks of any architecture in the domain of genomics.

### 3.2. Transfer Learning and Multitask Learning

The concept of transfer learning is naturally motivated by human intelligence whereby people can apply the knowledge that has already been acquired to address newly encountered problems. Transfer learning is such a framework that allows deep learning to adapt the previously trained model to exploit a new but relevant problem more effectively [123]. It has been successfully applied to other fields, such as language processing [124] or audio–visual recognition [125]. Readers could find surveys on transfer learning by Pan and Yang [126] or Weiss et al. [127]. Additionally, multitask learning is an approach that inductively shares knowledge among multiple tasks. By learning related tasks in parallel while using shared architectures, what is learned by a single task can be auxiliary to those related. An overview of multitask learning, which especially focuses on neural networks, can be found in Ruder [128]. Widmer and Rätsch [129] briefly discussed multitask learning from a biological perspective.

Early adaptation of transfer learning in genomics was based on machine learning methods such as SVMs [130,131,132]. Recent works have also involved deep learning. For example, Zhang et al. [133] developed a CNN model to analyze gene expression images for automatic controlled vocabulary (CV) term annotation. They pre-trained their model on ImageNet (http://www.image-net.org/ (accessed on 3 September 2023)) to extract general features at different scales and then fine-tuned the model by multitask learning to capture CV term-specific discriminative information. Liu et al. [134] developed an iterative PEDLA to predict enhancers across multiple human cells and tissues. They first pre-trained PEDLA on data derived from any cell type/tissue in an unsupervised manner and then iteratively fine-tuned the model on a subsequent cell type/tissue supervisedly, using the trained model of the previous cell type/tissue as initialization. Cohn et al. [135] transferred deep CNN parameters between networks trained on different species/datasets for enhancer identification. Qin and Feng [136] (TFImpute) adopted a CNN-based multitask learning setting to borrow information across TFs and cell lines to predict cell-specific TF binding for TF-cell line combinations from only a small portion of available ChIP-seq data. They were able to predict TFs in new cell types by models trained unsupervisedly on TFs where ChIP-seq data are available, which took the right step in the direction of developing a domain transfer model across cell types. Qi et al. [137] proposed a semi-supervised multitask framework for protein–protein interaction (PPI) predictions. They applied the MLP classifier trained supervisedly to perform an auxiliary task that leverages partially labeled examples. The loss of the auxiliary task is added to MLP loss so that the two tasks can be jointly optimized. Wang et al. [138] worked on the same problem by introducing a multitask convolutional network model for representation learning. Zhou and Troyanskaya [32] incorporated a multitask approach for noncoding-variant effects prediction on chromatin by jointly learning across diverse chromatin factors. Shao et al. [139] proposed a task relationship learning framework to automatically investigate the inherent correlation between diagnosis and prognosis genomics tasks, while DeepND [140] claimed to learn the shared and disorder-specific features using multitask learning setting where several tasks are solved together.

### 3.3. Multi-View Learning

As the current technology has made available data from multi-platform or multi-view inputs with heterogeneous feature sets, multi-view deep learning appears to be an encouraging direction for future deep learning research which exploits information across the datasets, capturing their high-level associations for prediction, clustering as well as handling incomplete data. Readers can visit Li et al. [141] for a survey on multi-view methods if interested. In many applications, we can approach the same problem from different types of data, such as in computer visions when audio and video data are both available [142,143]. Genomics is an area where data of various types can be assimilated naturally. For example, abundant types of genomic data (e.g., DNA methylation, gene expression, miRNA expression data) for the same set of tumor samples have been made available by the state-of-the-art high-throughput sequencing technologies [144]. Therefore, it is natural to think of leveraging multi-view information in genomics to achieve a better prediction than that of a single view. Gligorijević and Pržulj [145] and Li et al. [146] reviewed some methods for multi-view biological data integration with instructive considerations.

Multi-view learning can be achieved by, for example, concatenating features, ensemble methods, or multi-modal learning (selecting specific deep networks as sub-networks of the main model for each view and then integrating them in higher layers), just to name a few. The previously mentioned ultra-DNN [101] is a case in point, where it adopted 1D and 2D CNNs, respectively, for sequential features and spatial features. Liang et al. [144] proposed a multi-modal DBN to integrate gene expression, DNA methylation, miRNA, and drug response data to cluster cancer patients and define cancer subtypes. Their stacked Gaussian-restricted Boltzmann machines (gRBMs) are trained by contrastive divergence, different modalities are integrated via stacking hidden layers, and common features are effused from inherent features derived from multiple single modalities. In this direction, Zhang et al. [147] utilized a multi-modal DBN framework to integrate an RNA primary sequence with predicted secondary, and tertiary structures, while a later work Pan and Shen [39] went a step further with a hybrid multi-modal framework combining CNNs and DBNs to predict RNA-binding protein interaction sites and motifs on RNAs using five different modalities including region type, CLIP co-binding, structure, motif, and CNN sequence. Additionally, instead of dealing with different modalities separately, some research started to explore multi-modal interactions: for example, Shao et al. [139] performed an integrative analysis on histopathological image and genomic data for cancer diagnosis and prognosis, while Wang et al. [148] explored both intra-modality and inter-modality feature modules for genomic data and pathological images.

## 4. Genomic Applications

**Figure 1:**
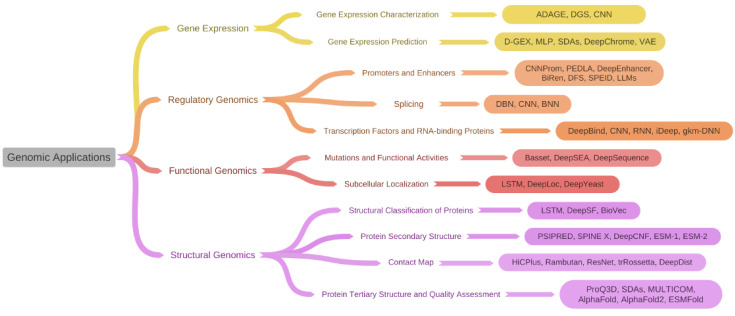
Taxonomy of genomic applications and corresponding deep learning models.

In this section, we review several aspects of genomic problems that can be approached from deep learning methods and discuss how deep learning moves forward in these fields. A summary of the taxonomy of different deep learning application areas with corresponding deep learning models discussed in this section can be found in Figure 1.

### 4.1. Gene Expression

Gene expression is a highly regulated process by which the genetic instructions in DNA are converted into functional products such as proteins and other molecules, and they also respond to the changing environment accordingly. Namely, genes encode protein synthesis and self-regulate the functions of the cell by adjusting the amount and type of proteins it produces [149]. Here, we review some research that applied deep learning to analyze how gene expression is regulated.

#### 4.1.1. Gene Expression Characterization

An increasing number of genome-wide gene expression assays for different species have become available in public databases; e.g., the Connectivity Map (CMap) project was launched to create a reference collection of gene expression profiles that can be used to identify functionally connected molecules [150]. These databases greatly facilitated the computational models for the biological interpretation of these data. At the same time, recent works have suggested better performance obtained by deep learning models on gene expression data; Urda et al. [151] used a deep learning approach to outperform LASSO in analyzing RNA-Seq gene expression profile data.

The empirical results of early works that applied principal component analysis (PCA) on gene expression data to capture cluster structure have shown that this mathematical tool was not effective enough to allow some complicated biological considerations [152]. Also, since the reliability of the cross-experiment datasets is limited by technical noise and unmatched experiment conditions [92], researchers are considering the denoising and enhancement of the available data instead of directly finding principal components.

Denoising autoencoders came in handy since they do not merely retain the information of raw data but also generalize meaningful and important properties of the input distribution across all input samples. Even shallow denoising autoencoders can be proven effective in extracting biological insights. Danaee et al. [153] adopted SDAs to detect functional features in breast cancer from gene expression profile data. Tan et al. [90] (ADAGE) presented an unsupervised approach that effectively applied SDA to capture key biological principles in breast cancer data. ADAGE is an open-source project for extracting relevant patterns from large-scale gene expression datasets. Tan et al. [91] further improved ADAGE to successfully extract both clinical and molecular features. To build better signatures that are more consistent with biological pathways and enhance model robustness, Tan et al. [92] developed an ensemble ADAGE (eADAGE) to integrate stable signatures across models. These three similar works were all experimented on Pseudomonas aeruginosa gene expression data. Additionally, Gupta et al. [89] demonstrated the efficacy of using the enhanced data by multilayer denoising autoencoders to cluster yeast expression microarrays into known modules representing cell cycle processes. Motivated by the hierarchical organization of yeast transcriptomic machinery, Chen et al. [154] adopted a four-layered autoencoder network with each layer accounting for a specific biological process in gene expression. This work also introduced sparsity into autoencoders. Edges of denoising autoencoders over PCA and independent component analysis (ICA) were clearly illustrated in the aforementioned works.

Some other works moved to variational inference in autoencoders, which is assumed to be more skillful at capturing the internal dependencies among data. Way and Greene [97] trained VAE-based models to reveal the underlying patterns in the pathways of gene expression and compared their three VAE architectures to other dimensionality reduction techniques, including the aforementioned ADAGE [90]. Dincer et al. [155] introduced the DeepProfile, a framework featuring VAE, to extract latent variables that are predictive for acute myeloid leukemia from expression data. Sharifi-Noghabi et al. [156] proposed Deep Genomic Signature (DGS), which is a pair of VAEs that are trained over unlabeled and labeled data separately from expression data for predicting metastasis.

Another thread for utilizing deep learning to characterize gene expression is to describe the pairwise relationship. Wang et al. [138] showed that CNN can be seen as an effective replacement for the frequently used Pearson correlation applied to pairs of genes; therefore, they built a multitask CNN that can consider the information of GO semantics and interaction between genes together to extract higher-level representations of gene pairs for the further classification task, which is further extended by two shared-parameter networks [157]. Recently, LLMs have come into play for such a pairwise relationship: Cui et al. [158] introduced a GPT-based foundational model and found a positive pairwise correlation between the similarity of the gene embeddings and the number of common pathways shared by these genes; similarly, Yang et al. [159] utilized the attention weights in transformers to reflect the contribution of each gene and the interaction of gene pairs.

#### 4.1.2. Gene Expression Prediction

Deep learning approaches for gene expression prediction have outperformed other existing algorithms. For example, Chen et al. [160] presented a three-layer feed-forward neural network for the gene expression prediction of selected landmark genes that achieved better performance than linear regression. This model, D-GEX, is of the multitask setting and was tested on two types of expression data, the microarrays and RNA-Seqs. Xie et al. [161] showed that their deep model based on MLP and SDAs outperformed Lasso and Random Forests in predicting gene expression quantifications from SNP genotypes.

When making predictions from gene sequences, deep learning models have been shown fruitful in identifying the context-specific roles of local DNA-sequence elements; then, the further inferred regulatory rules can be used to predict expression patterns [162]. Successful prediction usually relies heavily on the proper utilization of biological knowledge. Therefore, it could be more efficient to pre-analyze the contextual information in DNA sequences than directly make predictions. Deep learning models could refer to two early machine learning works that apply Bayesian networks to predict gene expression based on their learned motifs [162,163].

In most applications, the power of deep learning algorithms is impeded by biological restrictions. Therefore, instead of only using sequence information, combing epigenetic data into the model might add to the explanatory power of the model. For example, the correlation between histone modifications and gene regulation was suggested experimentally in Lim et al. [164], Cain et al. [165] and Dong and Weng [166], and it has already been studied in some machine learning works before [167,168,169,170]. Singh et al. [111] presented DeepChrome, a unified discriminative framework stacking an MLP on top of a CNN, and achieved an average AUC of 0.8 in a binary classification task that predicts high or low gene expression level. The input was separated into bins so as to discover the combinatorial interactions among different histone modification signals. The learned region representation is then fed into an MLP classifier that maps to gene expression levels. Additionally, Singh et al. [111] (DeepChrome) visualized a high-order combination to make the model interpretable. Other examples of epigenetic information that can be utilized in gene expression prediction tasks include DNA methylation, miRNA, chromatin features, etc.

Generative models were also adopted due to the ability to capture high-order, latent correlations. For example, to explore hypothetical gene expression profiles under various types of molecular and genetic perturbation, Way and Greene [54] trained a VAE on The Cancer Genome Atlas (TCGA; [171]) pan-cancer RNA-seq data to capture biologically relevant features. They have another previous work that evaluates VAEs of different architectures, which provided a comparison among VAEs, PCA, ICA, non-negative matrix factorization (NMF), and the aforementioned ADGAE [97]. Having the emergent capability to integrate long-range interactions in the genome, generative language models such as Avsec et al. [66] also claimed to improve gene expression prediction accuracy from DNA sequences, leading to more accurate variant effect predictions on gene expression for both natural genetic variants and saturation mutagenesis measured by massively parallel reporter assays.

### 4.2. Regulatory Genomics

Gene expression regulation is the cellular process that controls the expression level of gene products (RNA or protein) to be high or low. It increases the versatility of an organism so as to allow it to react and adapt to the surrounding environment. The underlying interdependencies behind the sequences limit the flexibility of conventional methods, but deep networks that could model an over-representation of sequence information have the potential to allow regulatory motifs to be identified according to their target sequences.

#### 4.2.1. Promoters and Enhancers

The most efficient way of gene expression regulation for an organism is at the transcriptional level, which occurs at the early stage of gene regulation. Enhancers and promoters are two of the most well-characterized types of functional elements in the regions of noncoding DNA, which belong to cis-regulatory elements (CREs). Readers can visit Wasserman and Sandelin [172] and Li et al. [173] for a review of early approaches for the identification of CREs.

Promoters locate near the transcription start sites of genes and thereby initiate the transcription of particular genes. Conventional algorithms still perform poorly on promoter prediction, while the prediction is always accompanied by a high false positive rate [174]. The compensation for sensitivity is usually achieved at the cost of specificity and renders the methods not accurate enough for applications. One initial work by Horton and Kanehisa [175] applied neural networks to predict E. coli promoter sites and provided a comparison of neural networks versus statistical methods. Matis et al. [176] also applied neural networks to promoter recognition, although it was assisted with some rules which use the gene context information predicted by GRAIL. These early works of deep learning models were not noticeable enough to demonstrate a clear edge over the weight matrix-matching methods. One recent study by Umarov and Solovyev [177] used a CNN with no more than three layers that well demonstrated the superiority of CNN over conventional methods in the promoter recognition of five distant organisms. Their trained model has been implemented as a web application called CNNProm. A more recent CNN-based model for enhancer prediction applied a transfer learning setting on different species/datasets [135]. Another highlight of their work lies in the design of adversarial training data.

PEDLA was developed by Liu et al. [134] as an algorithmic framework for enhancer prediction based on deep learning. It is able to directly learn from heterogeneous and class-imbalanced data and thus is an enhancer predictor that can be generalized across multiple cell types/tissues. The model has an embedded mechanism to handle class-imbalanced problems in which the prior probability of each class is directly approximated from the training data. PEDLA was first trained on nine types of data in H1 cells and then further extended with an iterative scheme that manages to generalize the predictor across various cell types/tissues. PEDLA was also compared with and outperformed some of the most typical methods for predicting enhancers.

Min et al. [33] (DeepEnhancer) adopted CNNs that surpass previous sequence-based SVM methods on the task of identifying enhancers from background genomic sequences. They compared different designs of CNNs and concluded the effectiveness of max-pooling and batch normalization for improving classification accuracy, while they also pointed out that simply increasing the depth of deep architectures is not useful if it is inappropriately designed. Their final model has been fine-tuned on ENCODE cell type-specific enhancer datasets from the model trained on the FANTOM5 permissive enhancer dataset by applying transfer learning.

Yang et al. [61] showed the possibility of predicting enhancers with DNA sequence alone with the presentation of BiRen, which is a hybrid of CNN and RNN. While demonstrating the possibility, there seems to be room to improve BiRen with the techniques that enable deep learning over heterogeneity data (e.g., see Section 5.1.3), since BiRen still exhibits weaker predictive performance than the methods that consider the cell-type-/tissue-specific enhancer markers explicitly.

Deep Feature Selection (DFS) is an attempt taken by Li et al. [178] to introduce sparsity to deep architectures. Conventionally, the sparseness is achieved by adding a regularization term (e.g., Lasso, Elastic Net). Li et al. [178] took a novel approach by which they can automatically select an active subset of features at the input level to reduce the feature dimension. This is implemented as an additional sparse one-to-one (point-wise product) linear layer between the input data and the input layer of the main model. DFS is widely applicable to different deep architectures. For example, Li et al. [178] demonstrated MLP-based DFS (shallow DFS) and DNNs-based DFS (Deep DFS), and they pointed out that when back-propagation does not perform well for deep networks, people can resort to stacked contractive autoencoder (ScA) and DBN-based DFS models that pre-trained layer-wisely in a greedy way before being fine-tuned by back-propagation. The author developed an open-source package of DFS and illustrated the superiority of DFS over Elastic Net and Random Forest in the identification of enhancers and promoters. Li et al. [179] further implemented a supervised deep learning package named DECRES, a feed-forward neural network based on DFS, for genome-wide detection of regulatory regions.

Enhancer–promoter interaction predictions are always based on non-sequence features from functional genomic signals. Singh et al. [59] (SPEID) proposed the first deep learning approach to infer enhancer–promoter interactions genome-wide from only sequence-based features as well as the locations of putative enhancers and promoters in a specific cell type. Their model was demonstrated to be superior to DeepFinder, which is based on machine learning [180]. This hybrid model consists of two parts. The first part accounts for the differences of underlying features that could be learned between enhancers and promoters and thus treats enhancers and promoters separately at input by two branches, where each branch is a one-layer CNN followed by a rectified linear unit (ReLU) activation layer. The second part is an LSTM that is responsible for identifying informative combinations of the extracted subsequence features. Their work provided insights into the long-range gene regulation determined from the sequences.

LLMs has made significant progress in promoter and enhancer-related prediction tasks. Ji et al. [63] utilized a BERT architecture to effectively predict proximal and core promoter regions, and the successor of this work [68] claimed to achieve optimal performance in the Core Promoter Detection task. Dalla-Torre et al. [104] performed comprehensive benchmarking on 17 datasets including predicting regulatory elements for enhancers and promoters for several transformer models.

#### 4.2.2. Splicing

Splicing refers to the editing of pre-messenger RNA so as to produce a mature messenger RNA (mRNA) that can be translated into a protein. This process effectively adds up to the diversity of protein isoforms. Predicting a “splicing code” aims to understand how splicing regulates and manifests the functional changes of proteins, and it is crucial for understanding different ways of how proteins are produced.

Initial machine learning attempts included the naive Bayes model [181] and two-layer Bayesian neural network (BNN) [182] that utilized over a thousand sequence-based features. Early applications of neural networks in regulatory genomics simply replaced a classical machine learning approach with a deep model. For example, Xiong et al. [183] adopted a fully connected feed-forward neural network trained on exon-skipping events in the genome that can predict splicing regulation for any mRNA sequence. They applied their model to analyze more than half a million mRNA-splicing codes for the human genome, and they discovered many new disease-causing candidates while thousands of known disease-causing mutations were successfully identified. This is a case where high performance mainly results from a proper data source rather than a descriptive model design. Lee and Yoon [184] presented a DBN-based approach that is capable of dealing with class-imbalanced data to predict splice sites while also identifying non-canonical splice sites. They also proposed a new training method called boosted contrastive divergence with categorical gradients and showed by their experiments its ability to improve prediction performance and shorten runtime compared to contrastive divergence or other methods. Gao et al. [185] developed an approach based on CNN and use sequence signatures to identify gene targets of a therapeutic for human splicing disorders.

In many cases, the phenomenon of alternative splicing occurs. That is, a single gene might end up coding for multiple unique proteins by varying the exon composition of the same mRNA during the splicing process. This is a key post-transcriptional regulatory mechanism that affects gene expression and contributes to proteomic diversity [186]. Leung et al. [187] developed a DNN model containing three hidden layers to predict alternative splicing patterns in individual tissues as well as across-tissue differences. The hidden variables of the model are designed to include cellular context (tissue types) information to extract genomic features. This is one of the initial works that adapts deep learning for splicing prediction. Jha et al. [188] used previously developed BNN [182] and DNN [187] models to design an integrative deep learning model for alternative splicing. They viewed previous work as the baseline on their original dataset and further developed these models by integrating additional types of experimental data (e.g., tissue type) and proposed a new target function. Their models are able to identify splicing regulators and their putative targets as well as infer the corresponding regulatory rules directly from the genomic sequence.

#### 4.2.3. Transcription Factors and RNA-Binding Proteins

Transcription factors (TFs) refer to proteins that bind to promoters and enhancers on DNA sequences and RNA-binding proteins, which—as the name suggested—are both crucial regulatory elements in biological processes. Current high-throughput sequencing techniques for selecting candidate binding targets for certain TFs are restricted by the low efficiency and high cost [189]. Researchers seeking computational approaches for TF binding sites prediction on DNA sequences initially utilized consensus sequences or their alternative, position weight matrices [190]. Later machine learning methods such as SVM using k-mer features [191,192] surpassed previous generative models.

Many existing deep learning methods approach TFBS prediction tasks through convolutional kernels. Alipanahi et al. [31] (DeepBind) have had success using CNN models in large-scale problems of TFBS tasks. Chen et al. [37] combined the advantage of representation learning from CNN and explicity reproduced kernel Hilbert space to introduce the convolutional kernel networks to predict TFBS with interpretability. Zeng et al. [34] conducted a systematic analysis of CNN architectures for predicting DNA sequence binding sites based on large TF datasets. Lanchantin et al. [58] further explored CNNs, RNNs, and the combination of the two in the task of TFBS with comprehensive discussion and visualization techniques. Admittedly, CNNs can sufficiently capture most sequential and spatial features in DNA sequences, but recurrent networks as well as bidirectional recurrent networks are useful when accounting for motifs in both directions of the sequence. Motivated by the symmetry of double-stranded DNA, which means that identical patterns may appear on one DNA strand and its reverse complement, Shrikumar et al. [193] proposed a traditional convolution-based model which shares parameters of forward and reverse-complement versions of the same DNA sequences, and they showed robust results on in vivo TFBS prediction tasks using chromatin ChIP-seq data. This is a novel work that tailors conventional neural networks to consider motifs through bidirectional characterizations.

In addition to convolutional neural networks, which proved powerful as long as they were appropriately designed according to the specific problem, some other approaches deal with different feature extraction or multiple data sources. Cross-source data usually share common knowledge at a higher abstraction level beyond the basic observation and thus need to be further integrated by the model. Zhang et al. [147] proposed a multi-modal deep belief network that is capable of the automatic extraction of structural features from RNA sequences; they first successfully introduce tertiary structural features of RNA sequences to improve the prediction of RNA-binding proteins’ interaction sites. Another multi-modal deep learning model for the same purpose was developed by Pan and Shen [39] (iDeep). This model consists of DBNs and CNNs to integrate lower-level representations extracted from different data sources. Cao and Zhang [194] (gkm-DNN) designed a model based on gapped k-mers frequency vectors (gkm-fvs) to extract informative features. The gkm-fvs after normalization is taken as input for a multilayer perceptron model trained by the standard error back-propagation algorithm and mini-batch stochastic gradient descent. By taking advantage of both gapped k-mer methods and deep learning, gkm-DNN achieved overall better performance compared with gkm-SVM. Qin and Feng [136] (TFImpute) proposed a CNN-based model that utilizes domain adaptation methods, which are discussed in more detail in Section 3.2, to predict TFs in new cell types by models trained unsupervisedly on TFs where ChIP-seq data are available. Ji et al. [63] and Zhou et al. [68] fine-tuned BERT-based models to more accurately predict TFBSs on both human and mouse genomic tracks.

### 4.3. Functional Genomics

#### 4.3.1. Mutations and Functional Activities

One of the shortcomings of previous approaches for predicting the functional activities from DNA sequences is the insufficient utilization of positional information. Although Ghandi et al. [191] upgraded the k-mer method by introducing an alternative gapped k-mers method (gkm-SVM), the improvement is not remarkable since the DNA sequence is still simply represented as vectors of k-mer counts without considering the position of each segment in the sequence. Although position-specific sequence kernels exist, they map the sequence into much higher dimension space and are thus not efficient enough [36].

In contrast to conventional methods, deep learning methods such as CNNs naturally account for positional relationships between sequence signals and are computationally efficient. Kelley et al. [36] (Basset) presented an open-source CNN-based package trained on the genomics data of 164 cell types and remarkably improved the prediction for functional activities of DNA sequences. Basset enables researchers to perform the single-sequencing assay and annotate mutations in the genome with present chromatin accessibility learned at the same time. Zhou and Troyanskaya [32] (DeepSEA) contributed another open-source deep convolutional network for predicting from only a genomic sequence the functional roles of noncoding variants on histone modifications, TFBS, and the DNA accessibility of sequences with high nucleotide resolution. Since CNN-based methods might require a certain amount of supervised training data, Benegas et al. [103] utilized pre-trained DNA language models to perform zero-shot non-coding variant effects prediction, and the results outperformed previous approaches where vast amounts of functional genomics data are required for training.

The effects of mutations are usually predicted by site-independent or pairwise models, but these approaches do not sufficiently model higher-order dependencies. Riesselman et al. [113] (DeepSequence) took a generative approach to track mutation effects that are beyond pairwise by biologically motivated Beyasian deep latent networks. They introduced latent variables on which DNA depends and visualized model parameters to illustrate the structural proximity and amino acid correlations captured by DeepSequence.

#### 4.3.2. Subcellular Localization

Subcellular localization is used to predict the subcellular compartment in a protein that resides in the cell from its biological sequence. In order to interact with each other, proteins need to at least temporarily inhabit physically adjacent compartments; therefore, the knowledge of protein location sheds light on where a protein might function as well as what other proteins it might interact with [195]. Most previous methods rely on SVMs and involve hand-generated features. For example, Shatkay et al. [195] (SherLoc) integrated different sequence and text-based features, and Pierleoni et al. [196] (BaCelLo) developed a hierarchy of binary SVMs. Meinken et al. [197] reported on previous tools and Wan and Mak [198] covered the machine learning approaches for subcellular localization. Some early deep learning works have shifted from SVMs to neural networks, such as Emanuelsson et al. [199] and Hawkins and Bodén [200]. Mooney et al. [201] used an N-to-1 neural network to develop a subcellular localization predictor (SCLpred). Sønderby et al. [48] adopted LSTM to predict protein subcellular locations from only sequence information with high accuracy. They further enhanced the model by adding convolutional filters before LSTM as a motif extractor and introducing the attention mechanism that forces the LSTM to focus on particular segments of the protein. The validity of their convolutional filters and attention mechanisms were visualized in experiments. Almagro Armenteros et al. [60] proposed a similar integrative hybrid model DeepLoc consisting of four modules, including a CNN, Bi-LSTM, an attention scheme and a q fully connected dense layer. Kobayashi et al. [202] utilized vector quantized VAE architecture to encode high-resolution features of protein subcellular localization without the need for prior knowledge, categories or annotations.

High-throughput microscopy images are a rich source of biological data that remains to be better exploited. One of the important utilizations of microscopy images is the automatic detection of the cellular compartment. Pärnamaa and Parts [117] (DeepYeast) devised an eleven-layer deep model for fluorescent protein subcellular localization classification in yeast cells, of which eight convolutional layers are succeeded by three fully connected layers. Internal outputs of the model are visualized and interpreted from the perspective of image characteristics. The author concluded that the low-level network functions as a basic image feature extractor, while higher layers account for separating localization classes.

### 4.4. Structural Genomics

#### 4.4.1. Structural Classification of Proteins

Proteins usually share structural similarities with other proteins, some of which have a common evolutionary origin [203]. The classification of protein structure can be traced back to the 1970s, aiming to comprehend the process of protein folding and protein structure evolution [204]. Grouping proteins into structural or functional categories also facilitates the understanding of an increasing amount of the newly sequenced genome.

Early methods for similarity measures mostly rely on sequence properties (i.e., they are alignment based), such as FASTA [205], BLAST [206], or PSI-BLAST [207], and they were then upgraded by leveraging profiles derived from multiple sequence alignments and position-specific scoring matrices (PSSMs) in addition to raw sequences [208] or discriminative models like SVM [209]. For example, Cang et al. [210] adopted SVM with a topological approach utilizing persistent homology to extract features for the classification of protein domains and superfamilies. Other top-performing deep learning works also rely on protein homology detection (one can visit Chen et al. [211] for a review) to deduce the 3D structure or function of a protein from its amino acid sequence. Hochreiter et al. [212] suggested a model-based approach that uses LSTM for homology detection. Their model makes similarity measures such as BLOSUM or PAM matrices not a priori fixed but instead suitably learned by LSTM with regard to each specific classification task. Liu et al. [51] (ProDec-BLSTM) conducted a similar work on protein remote homology detection and showed an improvement using Bi-LSTM instead of LSTM [212]. One drawback of homology-based approaches for fold recognition is the lack of a direct relationship between the protein sequence and the fold, since current methods substantially rely on the fold of the known template protein to classify the fold of new proteins [38]. Therefore, Hou et al. [38] (DeepSF) proposed a deep 1D CNN for fold classification directly from protein sequences.

There are also some works based on available gene function annotation vocabularies (e.g., Gene Ontology [213]) to perform protein classification [214]. As a result of similar motivation, BioVec [215] was designed as a deep learning method to compute a distributed representation of biological sequences with general genomic applications such as protein family classification. Each sequence is embedded in a high-dimension vector by BioVec, which reduces the classification of protein families to a simple classification task.

#### 4.4.2. Protein Secondary Structure

The protein Secondary Structure (SS) refers to the local spatial structure formed by the interaction between nearby stretches of a polypeptide chain. The protein SS encodes information for predicting the biophysical properties of amino acid residues, higher-level protein structures (e.g., tertiary structures), protein functions and evolution. It is traditionally described by either a three-state model [216] or an eight-state model by the DSSP algorithm [217]. The former labels each residue to be in one of three states: Helix, Strand, or Coil, while the eight-state model expands to eight different states for a more fine-grained description of the spatial environment and chemical bonding of each amino acid. Q3 and Q8 accuracies are the widely adopted metrics to evaluate any model performance, which represents the percentage of correctly predicted secondary conformation of amino acid residues. An alternative measure for three-state prediction is the segment of overlap (SOV) score [218]. The reasonable goal of SS prediction is suggested by Rost et al. [219] as a Q3 accuracy above 85%.

Before deep learning became popular for protein SS prediction, machine learning approaches including probabilistic graphical models [220,221,222], hidden Markov models [221] and SVMs [223,224,225] were widely adopted. In that nascent age of neural networks, one of the earliest applications developed a shallow feed-forward network that predicts protein SS and homology from the amino acid sequences [226]. Other works for SS prediction adopted similar or slightly enhanced neural networks [227,228]. Qian and Sejnowski [229] conducted one of the influential works for three-state prediction, reaching a Q3 accuracy of 64.3%. They then used the fully connected neural networks as a basis to develop a cascaded architecture, taking as the input window DNA sequences with orthogonal encoding. There was no significant progress for three-state prediction accuracy by neural networks until being improved to 70.8% by Rost and Sander [230,231]. Frustrated by the marginal influence of free parameters in the model, Rost and Sander [230] credited their improvement to leveraging evolutionary information encoded in the input profiles derived from multiple alignments. Riis and Krogh [232] achieved a practically identical performance by a structured neural network. They designed specific networks for each SS class according to biological knowledge, and the output prediction was made from filtering and ensemble averaging. Based on the PSSM generated by PSI-BLAST, Jones [233] (PSIPRED) used a two-stage neural network to obtain an average Q3 score of around 77%. Other popular deep learning methods such as bidirectional RNNs were also widely applied for protein SS prediction [234,235,236].

Emergent deep architectures for protein SS prediction have been widely explored with more prior knowledge and various features available. Faraggi et al. [237] (SPINE X) proposed an iterative six-step model, of which the neural network of each step follows a similar structure and is designed for each specific purpose. Spencer et al. [238] trained a deep belief network model in which an additional hidden layer is constructed to facilitate the unsupervised layer-by-layer initialization of the Restricted Boltzmann Machine (RBM). Li and Yu [239] designed a cascaded model, which leverages CNN to extract multi-scale local contextual features by different kernel sizes; then, they added a BRNN accounting for long-range dependencies in amino acid sequences to capture global contextual features.

Wang et al. [240] (DeepCNF) took a large step in improving Q3 accuracy above 80% by extending conditional neural fields (CDFs) to include convolutional designs. DeepCNF is able to capture both sequence–structure relationships and protein SS label correlation among adjacent residues. They also achieved Q8 accuracy of around 72%, outperforming the Q8 accuracy of 66.4% obtained by a supervised generative stochastic network [241]. Busia et al. [73] explored the model performance of eight-stated prediction from simple feed-forward networks to the adaptation of recent CNN architectures (e.g., Inception, ReSNet, and DenseNet). They modified the convolution operators of different scales and residual connections of successful CNN models in computer vision to suit the protein SS prediction task and also highlighted the differences compared to vision tasks. As opposed to the above-mentioned DeepCNF [240] that included interdependencies between labels of adjacent residues by a Conditional Random Field (CRF), Busia et al. [73] conditions the current prediction on previously predicted labels by sequence-to-sequence modeling.

A new class of Protein Language Model (ProtLM) has been proposed in recent years to utilize the power of large-scale, transformer-based language models. Rives et al. [64] introduced an ESM-1b model, which is a BERT-like model trained on up to 250 million protein sequence data from UniRef50 and UniRef100 datasets [242] using the common masked language model (MLM) objective, and they achieved 70%+ Q8 accuracies at family, superfamily, and fold levels on a constructed test set derived from the SCOPe database [243]. The ProtTrans model family [65] introduced a series of transformer model architectures (Transformer-XL [244], BERT [102], and T5 [245]) pre-trained on protein sequences from UniRef [242] and the Big Fantastic Database [246]. They also showed the potential of ProtLM completely independent of multiple sequence alignments (MSAs) features. Their best-performing model achieved a Q3 accuracy of 74.1% and Q8 accuracy of 60.7% on CASP14 [247]. The ESM-2 model family [71] was later introduced as an upgrade of the ESM-1b model in size (up to 15 billion parameters) and pushed the Q3 accuracy to 76.8% and Q8 accuracy to 61.7% on CASP14. The latest effort in scaling up ProtLM has resulted in a 100-billion-parameter model, xTrimoPGLM [72], with a General Language Model (GLM) as the model backbone [248]; however, the authors also pointed out that the logarithmic increase of model performance on size has already shown saturation on specific tasks such as the Q3 SS prediction.

#### 4.4.3. Contact Map

A protein contact map is a binary 2D matrix denoting the spatial closeness of any two residues in the folded 3D protein structure. Predicting residue–residue contact is thus crucial to protein structure prediction, and it has been studied using shallow neural networks [249]. Recent works proceeded to deeper networks. Lena et al. [99] stacked together multiple standard three-layer feed-forward networks sharing the same topology, taking into consideration both spatial and temporal features to predict protein residue–residue contact. Wang et al. [101] also developed an ultra-deep model to predict protein contacts from amino acids sequence. Their model consists of two deep residual neural networks that process 1D and 2D features separately and subsequently in order to consider both sequential and pairwise features in the whole model. Zhang et al. [41] and Schreiber et al. [40] both contributed an open-source multi-modal CNN model for Hi-C contact map prediction. Zhang et al. [41] (HiCPlus) first interpolated the low-resolution Hi-C matrix to the size of the high and then trained their model to predict a high-resolution matrix from the low-resolution matrix. The final output was recombined into the entire Hi-C interaction matrix. Schreiber et al. [40] (Rambutan) predicted Hi-C contacts at high resolution (1 kb) from nucleotide sequences and DNaseI assay signal data. Their model consists of two arms with each arm processing one type of data independently. The learned feature maps are then concatenated for further combination with genomic distance in the dense layers. Adhikari et al. [42] proposed a two-layer CNN network that consumes PSSM-based features as well as coevolutionary contact features to classify residue–residue contact into five distance bins (6–10 Å).

In recent years, ResNet [250] has been extensively used in contact map prediction. Yang et al. [46] proposed trRossetta (based on Rossetta3 software [251]), which utilized known MSAs to guide the training of a 60-layer ResNet to classify inter-residue distance as well as inter-residue orientational angles into discrete value bins. DeepDist [47] showcased the potential of ResNet for the direct prediction of residue–residue contact distance by combining outputs from four ResNet networks, each on one type of sequential or co-evolutional features from the input, and it was trained successfully with a regression objective that minimizes the mean squared error (MSE) of predicted contact distance in absolute value.

#### 4.4.4. Protein Tertiary Structure and Quality Assessment

The prediction of protein tertiary structure has proven crucial to human understanding of protein functions [252] and can be applied to, for instance, drug designs [253]. However, experimental methods for determining protein structures, such as X-ray crystallography, are costly and sometimes impractical. Although the number of experimentally solved protein structures included in the protein data bank (PDB) (https://www.rcsb.org/ (accessed on 10 September 2023)) keeps growing, it only accounts for a small proportion of currently sequenced proteins [254]. Thus, a potentially practical approach to fill the gap between the number of known protein sequences and the number of found protein structures is through computational modeling.

Two essential challenges in protein structure prediction include the sampling and the ranking of protein structural models [255]. Quality assessment (QA) is used to predict the absolute or relative quality of the protein models before the native structure is available so as to rank them. Some previous research, such as ([256], ProQ2) and ([257], ProQ3), was conducted based on machine learning models. Recent deep learning-based work from Uziela et al. [258] (ProQ3D) achieved substantial improvement by replacing the SVMs in previous work with DNNs. As opposed to these existing methods that rely on energy or scoring functions, Nguyen et al. [259] based solely on geometry to propose a sparse stacked autoencoder classifier that utilizes the contact map. Another research by Cao et al. [260] adopted a deep belief network protein structure prediction. Their model could be used to evaluate the quality of any protein decoy. Local quality assessment remains to be substantially improved compared with global prediction [261]. Liu et al. [262] introduced three models based on SDAs as a benchmark of deep learning methods for assessing the quality of individual protein models.

Modern tertiary structure prediction systems typically pipeline functional modules that (i) query MSA for target protein sequence; (ii) predict the contact map or residue–residue distance, and (iii) reconstruct a 3D structure based on predicted contact map under energy and physical constraints. The quality of these MSA-based systems can depend sensitively on the performance of the involved contact map prediction models (Section 4.4.3). MULTICOM [74] extended the use of DNCON2 as their contact map prediction module by incorporating 1D structural features, such as residue-level secondary structure labels, sequential features, and co-evolutionary features; the system was upgraded in 2022 to MULTICOM2 [78] as the authors incorporated more deep learning-based modules, including using DeepDist in place of DNCON2 for contact map prediction, and achieved high system ranking (seventh out of 146 systems) in the tertiary structure prediction in CASP14. ThreadAI [76] also improved upon MULTICOM by adopting trRossetta instead of DNCON2 in their contact map prediction.

The AlphaFold [75] and AlphaFold2 models [77] revolutionized the practicality of DNNs in predicting protein structure at atomic resolution: the authors constructed a novel two-stage DNN relying heavily on the attention mechanism to predict directly the 3D coordinates of all heavy atoms in a given protein: the first stage encodes and combines both MSA and residue pairs features through a series of transformer-like blocks with attention module; the second stage module then builds upon the learned representations to refine a hypothesized 3D structure subjecting to evolutionary, physical, and geometrical constraints. As of the writing of this review, AlphaFold2 remains the best model for protein structure prediction on CASP14. Research since has revealed several limitations of the AlphaFold2 model: its performance could suffer from predicting intrinsically disordered proteins Ruff and Pappu [263]; the performance on loop prediction is only high for short loops Stevens and He [264]; and significant degradation in performance was discovered on a target sequence with few homologous counterparts in existing databases or when the MSAs are of low depth Wang et al. [265], although this problem was mitigated by MSA-Augmenter Zhang et al. [266], which is a transformer model trained on known MSA sequences to generate artificial MSA sequences that are used to augment training data used by AlphaFold2.

A more serious limitation with AlphaFold2 is the inability of predicting novel structures due to its dependence on known MSA features. ProtLM models which are independent of MSA features overcome this limitation trivially and have shown great potential in novel structure prediction. Lin et al. [71] introduced ESMFold as an extension of the ESM-2 model family with an added folding structure module; ESMFold depends only on embeddings learned through ESM-2 and showed decent performance (80% of AlphaFold2) on CASP14. More competitive performance is achieved by newer ProtLM such as OmegaFold [67] and EMBER3D [69]. These models also showed much better inference time than AlphaFold2, with OmegaFold attaining sub-second prediction on proteins with sequence up to 1000 residues.

## 5. Challenges and Opportunities

With the discussion of the successes of applications of deep learning in genomics, now we proceed to discuss some current challenges. As deep learning models are usually over-parametrized, the performance can be conditional if the models are not appropriately designed according to the problem. There are multiple worthwhile considerations and techniques involving model architectures, feature extraction, data limitation, etc., which help deep learning models better approach genomics. Here, we briefly discuss some current challenges that deserve attention and several potential research directions that might shed on light the future development of deep learning applications in genomic research.

### 5.1. The Nature of Data

An inevitable challenge of transferring the success of deep learning in conventional vision or text data into genomics is raised due to the nature of the genomic data, such as the unavailability of true labels due to the lack of knowledge of the genetic process, the imbalanced case and control samples due to the rarity of a certain disease, and the heterogeneity of data due to the expensiveness of large-scale data collection. While privacy is always a notable challenge in genomic data sharing and research [267], dealing with complex, class-imbalanced, or heterogeneous data at a large scale requires a closer look into each scenario discussed below [268].

#### 5.1.1. Class-Imbalanced Data

Large-scale biological data gathered from assorted sources are usually inherently class imbalanced. Take epigenetic datasets, for example: there are in nature much fewer DNA methylated region (DMR) sites than non-DMR sites [269]. It is also common in enhancer prediction problems where the number of non-enhancer classes overwhelmingly exceeds that of enhancer classes [270,271]. This is problematic in the training of neural networks because the majority class is dominating the cost function and the network may simply learn to output the majority class every time and still achieve a decent accuracy, so the evaluation of precision and recall needs to be considered carefully [272]. Methods that directly oversample minor classes or undersample majority classes have been attempted and proven successful. Öztornaci et al. [273] found that multiple machine learning models (SVM, MLP, Random Forest) benefit from the Synthetic Minority Oversampling Technique (SMOTE) in finding single nucleotide polymorphisms (SNPs). This data-imbalance issue has also been encountered in machine learning methods [274,275], while ensemble methods appear to be powerful [269]. Sun et al. [276] applied the undersampling method together with a majority vote to address the imbalanced data distribution inherent in gene expression image annotation tasks. In deep learning approaches, Al-Stouhi and Reddy [277] based on boosting to propose an instance-transfer model to reduce the class-imbalanced influence while also improving the performance by leveraging data from an auxiliary domain. Combining conventional DNNs with a Deep Decision Tree classifier, R. et al. [278] proposes a Hybrid DNN architecture which addresses the class imbalance of certain RNA sequences by forcing the network to account for minor classes in the decision tree’s hierarchical if–else cases. In addition to resorting to ensemble approaches, researchers can manage to resolve class-imbalanced problems through model parameters or training processes. For instance, Liu et al. [134] (PEDLA) used an embedded mechanism utilizing the prior probability of each class directly estimated from the training data to compensate for the imbalance of classes. Lee and Yoon [184] presented a method called boosted contrastive divergence with categorical gradients for training RBMs for the class-imbalanced prediction of splice junctions. Singh et al. [59] performed data augmentation by slightly shifting each positive promoter or enhancer within the window since the true label is not sensitive to these minimal changes. They also designed the training procedure accordingly to avoid the high false positive rate resulting from the augmented dataset.

#### 5.1.2. Various Data Types

Intuitively, integrating diverse types of data as discriminating features will lead to more predictive power of the models. For example, Liu et al. [134] (PEDLA) trained their model on nine types of data to identify enhancers, including chromatin accessibility (DNase-sseq), TFs and cofactors (ChIP-seq), histone modifications (ChIP-seq), transcription (RNA-Seq), DNA methylation (RRBS), sequence signatures, evolutionary conservation, CpG islands, and the occupancy of TFBSs, resulting in better model performance in terms of multiple metrics compared with existing popular methods. Angermueller et al. [100] (DeepCpG) predicted single-cell DNA methylation states by two disparate sub-networks designed accordingly for CpG sites and DNA sequences.

It pays off to manage to utilize the data of multiple views; although merging the information from various data sources challenges the models that could sufficiently integrate them, this effort might provide more information with a great chance. Also, critical feature processing has proven helpful to facilitate data integration [279]. A review of data representations in genomics, transcriptomics, proteomics, metabolomics and epigenomics for computer scientists can be found in Tsimenidis et al. [280], in which the format, type, and encoding of data from these disciplines are presented together with their common feature extraction techniques. For more discussions on encompassing diverse data sources, we refer to multi-view learning in Section 3.3.

#### 5.1.3. Heterogeneity and Confounding Correlations

The data in most genomic applications involving medical or clinical experiments are heterogeneous due to population subgroups or regional environments. One of the problems of integrating these different types of data is the underlying interdependencies among these heterogeneous data. Covariance is sometimes confounding and renders the model prediction inaccurate.

The Genome-Wide Association Study (GWAS) is an example where both population-based confounders (population subgroups with different ancestry) and individual relateness produce spurious correlations among SNPs to the trait of interest. The GWAS investigates the entire genome to identify SNPs associated with diseases or traits of interest, enabling people to predict the genetic predisposition of a disease and cure it by targeting corresponding SNPs. The challenges are twofold: one is to develop more expressive casual models, and the other is to address confounding factors across heterogeneous groups [281]. Most existing statistical methods estimate confounders before performing causal inference. These methods are based on linear regression [282,283], the linear mixed model (LMM) [284,285], or others [286]. Wang et al. [287] tried to upgrade LMM and tested it on biological variable selection and prediction tasks. Although these LMM-based models (e.g., FaST-LMM, [288]) are favored by some researchers and mathematically sufficient, their power pales when faced with multiple nonlinear confounding correlations. The assumed Gaussian noise might overshadow the true underlying causality, and LMM also fails to literally model the variable correlations. A seemingly more reliable approach is through generative modeling, e.g., Hao et al. [289]. Tran and Blei [290] and Louizos et al. [291] are all based on variational inference to present an implicit causal model for encoding complex, nonlinear causal relationships, taking into account latent confounders. Tran and Blei [290] optimized their model iteratively to estimate confounders and SNPs, and their simulation study suggested a significant improvement.

From the methodology perspective, several deep learning methods that are not designed exclusively for confounder correction, such as the domain adversarial learning [292], select-additive learning [143], and confounder filtering [293], can be reused once the identification of confounder is presented.

### 5.2. Feature Extraction

Deep learning that performs automatic feature extraction saves great efforts in choosing hand-engineered features. With the vast scale of typical genomics data and the complexity of sequences, manual features, such as the GC content of a DNA sequence, carry little distinguishable information compared to automatic feature extraction powered by deep learning, which is able to interpret more subtle features from the data [294].Torng and Altman [295] also discussed the superiority of automatically generated features over manually selected features. However, in practice, it is unfortunately time consuming to directly learn features from genomic sequences when complex interdependences and long-range interactions are taken into consideration. Researchers might still resort to task-specific feature extraction before automatic feature detection, which could strongly facilitate the model if skillfully designed.

#### 5.2.1. Mathematical Feature Extraction

Although deep neural networks are capable of capturing high-level features from low-level input data, the entangled geometric complexity and biological complexity underlying the 3D biomolecular structure of the protein have greatly hindered the generalization of deep learning to tremendous biological problems [296]. Techniques borrowed from mathematics have great potential to interpret the complex biological structures behind data. For example, topology is a promising choice to untangle the geometric complexity underlying the 3D biomolecular structure of proteins [296], and homology detection has been widely applied to protein classification problems [210,212]. Dey and Mandal [297] showed how protein classification can be improved by topological features. DeepMethyl [298] was developed as deep learning software using features derived from 3D genome topology and DNA sequence patterns. It is based on SDAs and is applied to predict methylation states of DNA CpG dinucleotides. Cang and Wei [296] introduced element-specific persistent homology (ESPH) into CNNs to predict protein-ligand binding affinities and protein stability changes upon mutation, including globular protein mutation impacts and membrane protein mutation impacts. Finally, to make feature extraction techniques from the popular literature generally available to the genomics research community, Bonidia et al. [299] made available a novel software package *MathFeature*, which implements 20 mathematical descriptors and 17 convential descriptors used to numerically encode long gene sequences.

#### 5.2.2. Feature Representation

By the conceptual analogy of the fact that humans communicate through languages, biological organisms convey information within and between cells through information encoded in biological sequences. To understand this language of life, Asgari and Mofrad [215] designed BioVec, an unsupervised data-driven feature representation method, which embeds each trigram of biological sequence in a 100-dimensional vector that characterizes biophysical and biochemical properties of sequences. BioVec was trained by a variant of MLP adapted from word2vec [112,300], which is a typical method in NLP. Following this work, Jin et al. [301] applies BioVec in the gene sequence encoding to predict long noncoding RNA protein (lncRNA-protein) iterations using Graph Autoencoders. Ng [302] further utilized shallow two-layer neural networks to compute the representation of variable-length k-mers of DNA sequences that is consistent across different lengths. In contrast to representation by BioVec for individual kmers, Kimothi et al. [303] built on the doc2vec algorithm (an extension of word2vec) to propose the distributed representation of a complete protein sequence and successfully applied it to protein classification following the settings of Asgari and Mofrad [215].

Another feature representation technique was proposed by Hao et al. [304] on cancer survival prediction from genetic sequences. The authors specifically represented the shared and unique features from DNA, mRNA and miRNA, and they leveraged the consistent and complement information in these features to improve prediction accuracy. These types of feature representation have the potential to facilitate future genomics research.

## 6. Conclusions and Outlook

Genomics is a challenging application area of deep learning that encounters unique challenges compared to other fields such as vision, audio, and text processing, since we have limited abilities to interpret genomic information but expect from deep learning a superhuman intelligence that explores beyond our knowledge. Yet deep learning is undoubtedly an auspicious direction that has constantly rejuvenated and moved forward genomic research in recent years. As discussed in this review, recent breakthroughs of deep learning applications in genomics have surpassed many previous state-of-the-art computational methods with regard to predictive performance, although they slightly lag behind traditional statistical inferences in terms of interpretation.

Current applications, however, have not brought about a watershed revolution in genomic research. The predictive performances in most problems have not reached the expectation for real-world applications and neither have the interpretations of these abstruse models elucidate insightful knowledge. A plethora of new deep learning methods are constantly being proposed but await artful applications in genomics. By the careful selection of data sources and features or the appropriate design of model structures, deep learning can be driven toward a bright direction which produces a more accurate and interpretable prediction. We need to bear in mind numerous challenges beyond simply improving predictive accuracy to seek essential advancements and revolutions in deep learning for genomics.

## Figures and Tables

**Table 1:** Overview of deep neural network architectures and their application in genomics. “Multiple” means multiple architectures have been studied in the references. Refer to Section 2 for technical details on each architecture.

Architecture	Reference	Year	Application in Genomics
CNN	Alipanahi et al. [31]	2015	Protein—binding
	Zhou and Troyanskaya [32]	2015	DNA sequence—noncoding variants
	Min et al. [33]	2016	DNA sequence—enhancers
	Zeng et al. [34]	2016	Protein—binding
	Lanchantin et al. [35]	2016	Protein— TFBS classification
	Kelley et al. [36]	2016	DNA sequence—functional activities
	Chen et al. [37]	2017	Protein—TFBS classification
	Hou et al. [38]	2017	Protein—fold classification
	Pan and Shen [39]	2017	Protein—RNA binding
	Schreiber et al. [40]	2017	Protein—contact map prediction
	Zhang et al. [41]	2017	Protein—contact map prediction
	Adhikari et al. [42]	2018	Protein—contact map prediction
	Kelley et al. [43]	2018	DNA sequence—phenotype to genotype prediction
	Xuan et al. [44]	2019	RNA sequence—noncoding genes
	Kelley [45]	2020	DNA sequence—gene regulation
	Yang et al. [46]	2020	Protein—inter-residue distance prediction
	Wu et al. [47]	2021	Protein—inter-residue distance prediction
RNN	Sønderby et al. [48]	2015	Protein—subcellular localization
	Quang and Xie [49]	2016	DNA sequence—noncoding function
	Cao et al. [50]	2017	Protein—function prediction
	Liu et al. [51], ProDec-BLSTM	2017	Protein—remote homology detection
	Boža et al. [52]	2017	DNA/RNA sequence—nanopore base calling
	Singh et al. [53]	2020	Protein—RNA binding
VAE	Way and Greene [54]	2017	Cancer—cancer gene expression
	Choi and Chae [55]	2020	DNA—methylome dataset construction
	Rashid et al. [56]	2021	Cancer—unmasking tumor heterogeneity
	Nissen et al. [57]	2021	Cancer—metagenomic binning
Hybrid	Sønderby et al. [48]	2015	Protein—subcellular localization
	Quang and Xie [49]	2016	DNA sequence—noncoding variants prediction
	Lanchantin et al. [58]	2016	Protein—TFBS classification
	Singh et al. [59]	2016	DNA sequence—enhancer promoter interaction
	Almagro Armenteros et al. [60]	2017	Protein—subcellular localization
	Yang et al. [61]	2017	DNA sequence—enhancers
	Li et al. [62]	2021	DNA sequence—regulatory function
Transformer	Ji et al. [63]	2017	DNA sequence—core promoter detection
	Rives et al. [64]	2019	Protein—ProtLM; secondary structure
	Elnaggar et al. [65]	2020	Protein—ProtLM; secondary structure; tertiary structure
	Avsec et al. [66]	2021	DNA sequence—gene expression prediction
	Wu et al. [67]	2022	Protein—ProtLM; secondary structure; tertiary structure
	Zhou et al. [68]	2023	DNA sequence—core promoter detection; Protein—TFBS classification
	Weissenow et al. [69]	2023	Protein—ProtLM; secondary structure; tertiary structure
	Nguyen et al. [70]	2023	Genomic language model
	Lin et al. [71]	2023	Protein—ProtLM; secondary structure; tertiary structure
	Chen et al. [72]	2023	Protein—ProtLM
Multiple	Busia et al. [73]	2016	Protein—secondary structure
	Hou et al. [74]	2019	Protein—contact map; tertiary structure
	Senior et al. [75], AlphaFold	2020	Protein—secondary structure; tertiary structure
	Zhang and Shen [76]	2020	Protein—contact map; tertiary structure
	Jumper et al. [77], AlphaFold2	2021	Protein—secondary structure; tertiary structure
	Liu et al. [78]	2022	Protein—contact map; tertiary structure

## Data Availability

No new data were created.
